# Internet Use and Higher-Level Functional Capacity Decline Suppression in Japanese Older Adults With Low Education: JAGES 2016-2019 Longitudinal Study

**DOI:** 10.2196/53384

**Published:** 2024-09-20

**Authors:** Atsuko Tajika, Atsushi Nakagomi, Yasuhiro Miyaguni, Chie Koga, Katsunori Kondo, Toshiyuki Ojima

**Affiliations:** 1 Department of Community Health and Preventive Medicine Hamamatsu University School of Medicine Shizuoka Japan; 2 Department of Social Preventive Medical Sciences Center for Preventive Medical Sciences Chiba University Chiba Japan; 3 Nihon Fukushi University Faculty of Social Welfare Aichi Japan; 4 Research Center for Advanced Science and Technology The University of Tokyo Tokyo Japan; 5 Department of Gerontological Evaluation Center for Gerontology and Social Science National Center for Geriatrics and Gerontology Aichi Japan

**Keywords:** functional capacity, instrumental activities of daily living, older adults’ cognitive engagement, older adults’ social role, internet impact on seniors, educational attainment, low education, independent living, older adults, health disparities

## Abstract

**Background:**

Higher-level functional capacity (HLFC) is crucial for the independent living of older adults. While internet use positively impacts the health of older adults, its effect on HLFC and how this effect varies with educational attainment remains uncertain.

**Objective:**

This longitudinal study aimed to investigate whether internet use could mitigate the risk of HLFC decline and if this benefit extends to older adults with lower levels of education.

**Methods:**

The data were sourced from the Japan Gerontological Evaluation Study (JAGES), encompassing 8050 community-dwelling adults aged 65 years and older from 2016 to 2019. The study focused on those who remained self-sufficient from 2016 to 2019, identifying participants with independent HLFC in 2016. The Tokyo Metropolitan Institute of Gerontology Index of Competence defined HLFC operationally, consisting of 3 subscales, namely instrumental activities of daily living, intellectual activity, and social role. The primary variable was the frequency of internet use in 2016; participants who reported using the internet were classified as internet users, while those who answered “No” were identified as nonusers. The study compared the effects of internet use on HLFC decline across educational levels of ≤9 years, 10-12 years, and ≥13 years using Poisson regression analysis adjusted for robust SE to calculate the risk ratio (RR) and 95% CI for HLFC decline in 2019.

**Results:**

After adjusting for demographic and health condition risk factors, internet use was significantly linked to a decreased risk of HLFC decline in older adults over 3 years, including those with lower educational levels. Internet users with ≤9 years of educational attainment experienced a suppressed decline in the total score (RR 0.57, 95% CI 0.43-0.76; *P*<.001); instrumental activities of daily living (RR 0.58, 95% CI 0.38-0.91; *P*=.02), intellectual activity (RR 0.60, 95% CI 0.41-0.89; *P*=.01), and social role (RR 0.74, 95% CI 0.56-0.97; *P=*.03) compared with nonusers. Participants with 10-12 years of education showed suppression rates of 0.78 (95% CI 0.63-0.98; *P=*.03), 0.59 (95% CI 0.39-0.90; *P*=.01), 0.91 (95% CI 0.63-1.31; *P=*.61), and 0.82 (95% CI 0.68-1.00; *P*=.05), respectively, and those with ≥13 years displayed suppression rates of 0.65 (95% CI 0.51-0.85; *P*=.001), 0.55 (95% CI 0.36-0.83; *P*=.01), 0.64 (95% CI 0.37-1.10; *P=*.11), and 0.83 (95% CI 0.64-1.08; *P=*.17), respectively.

**Conclusions:**

These findings indicate that internet use supports the maintenance of HLFC independence in older adults with higher education and those with lower educational levels. Encouraging internet use among older adults with lower levels of education through future policies could help narrow functional health disparities associated with educational attainment.

## Introduction

### Maintaining Higher-Level Functional Capacity Is Essential for the Independent Living of Older Adults

The World Health Organization Scientific Group on the Epidemiology of Aging suggests using autonomy or independence in life functioning as a health index for the older adult demographic [1]. In Japan, over 70% of older adults express a desire to continue residing in familiar surroundings and at home; they prefer to receive nursing care services at their residence, even if such care becomes necessary [2]. The Ministry of Health, Labour, and Welfare is advocating for the development of a comprehensive community support and service system known as the community-based integrated care system. This initiative aims to uphold the dignity of older adults and support their independent living within their local communities throughout their lives [3]. Nonetheless, even with this system, the gap between average and healthy life expectancy, referred to as the restriction period for daily life, stands at 8.7 years for men and 12.1 years for women [4].

Lawton [5] defined and systematized 7 hierarchical competencies for older adults: life maintenance, functional health, perception and cognition, physical self-maintenance (corresponding to basic activities of daily living), instrumental self-maintenance, effectance, and social role in the order of increasing complexity. “Instrumental self-maintenance” corresponds to instrumental activities of daily living (IADL) as the capability to maintain one’s life independently at home. “Effectance” is associated with engagement in intellectual activities, such as leisure and creativity, and “social role” corresponds to the ability to engage in intimate societal interactions. A study analyzing a nationally representative sample in Japan revealed that most community-dwelling older adults exhibit proficient functional capacity (eg, IADL, intellectual activity, and social role), although this capacity tends to diminish with age [6] and often deteriorates before declines in basic activities of daily living are observed [7]. Further research indicates that, after retirement, older adults aged ≥65 years’ experience a gradual decline in their higher-level functional capacity (HLFC; IADL, intellectual activity, and social role) [8] and that HLFC is linked to mortality as well as medical and long-term care expenses [9,10]. Consequently, HLFC emerges as a modifiable health factor vital for sustaining independent living among older adults, highlighting the importance of preventing HLFC loss.

### Addressing the Impact of Educational Disparities on HLFC Maintenance Is Imperative for All Older Adults

The capacities of older adults are likely influenced by the cumulative effect of health inequalities, which are exacerbated by factors, such as gender and education level, over the lifespan [11]. Longitudinal research in Japan has shown a significant correlation between HLFC and educational achievement [6], with additional studies indicating notably poor IADL among older adults with lower levels of education [12-15]. To facilitate independent living across this demographic, it is crucial to propose intervention strategies aimed at mitigating the decline in HLFC, including measures to enhance IADL capabilities, irrespective of educational background. Despite this need, established strategies for enabling older adults to decelerate the HLFC decline, especially among those with minimal educational attainment, remain scarce.

### The Internet May Have a Positive Effect on the HLFC of Older Adults

In Japan, the number of older adults using the internet in the past year has risen by 10.5% among those aged 60-69 years, 12.7% among those aged 70-79 years, and 7.5% among those aged 80 years and older, compared with 4 years earlier [16]. The spread of information and communication technology (ICT) among older adults is not limited to Japan but is also observed globally [17]. ICT has been demonstrated to yield numerous health benefits [18-39]. However, the relationship between internet use and HLFC has been the subject of limited research. Digital illiteracy, often attributed to a lack of education, emerges as a principal factor behind the digital divide, particularly among older populations [40]. Consequently, the effectiveness of the Internet in enhancing HLFC may be diminished for older adults with lower educational levels. Nevertheless, from the perspective of addressing health disparities, it is crucial to ascertain whether the internet can play a role in preventing HLFC loss among older adults with minimal education.

### Aims of This Study

To ensure that all older adults, irrespective of educational background, can maintain independent living, this study seeks to examine whether internet use can mitigate the risk of HLFC decline. In addition, it investigates whether such benefits extend to older adults with limited education. Until now, no research has explored the relationship between internet use and HLFC across different levels of educational attainment. This study aims to determine whether internet use can reduce the risk of HLFC decline among older adults with lower educational backgrounds, focusing on 3 subscales, which are “IADL,” “intellectual activity,” and “social role,” by categorizing individuals aged ≥65 years based on their educational levels.

## Methods

### Design, Setting, and Study Participants

Data were collected from the Japan Gerontological Evaluation Study (JAGES), a triennial community-based survey tracking older adults in Japan [41]. Longitudinal panel data from surveys conducted in 2016 and 2019 were used. The baseline survey took place between September 2016 and January 2017, with self-reported questionnaires dispatched to 34,571 independent community-dwelling individuals aged ≥65 years. Of these, 24,313 responded (response rate 70.3%).

The follow-up survey was carried out between November 2019 and January 2020. From the 24,313 baseline respondents, 8077 participated in the follow-up survey (response rate 33.2%). We excluded 27 responses due to discrepancies in reported age (n=26) and sex (n=1) between the baseline and follow-up surveys, leaving 8050 participants for analysis. In Japan, 81.6% of older adults in the community live independently [4]. Given the Japanese long-term care prevention project’s goal to avert HLFC decline among community-dwelling older adults, this demographic was deemed the most appropriate for the study.

**Figure 1:**
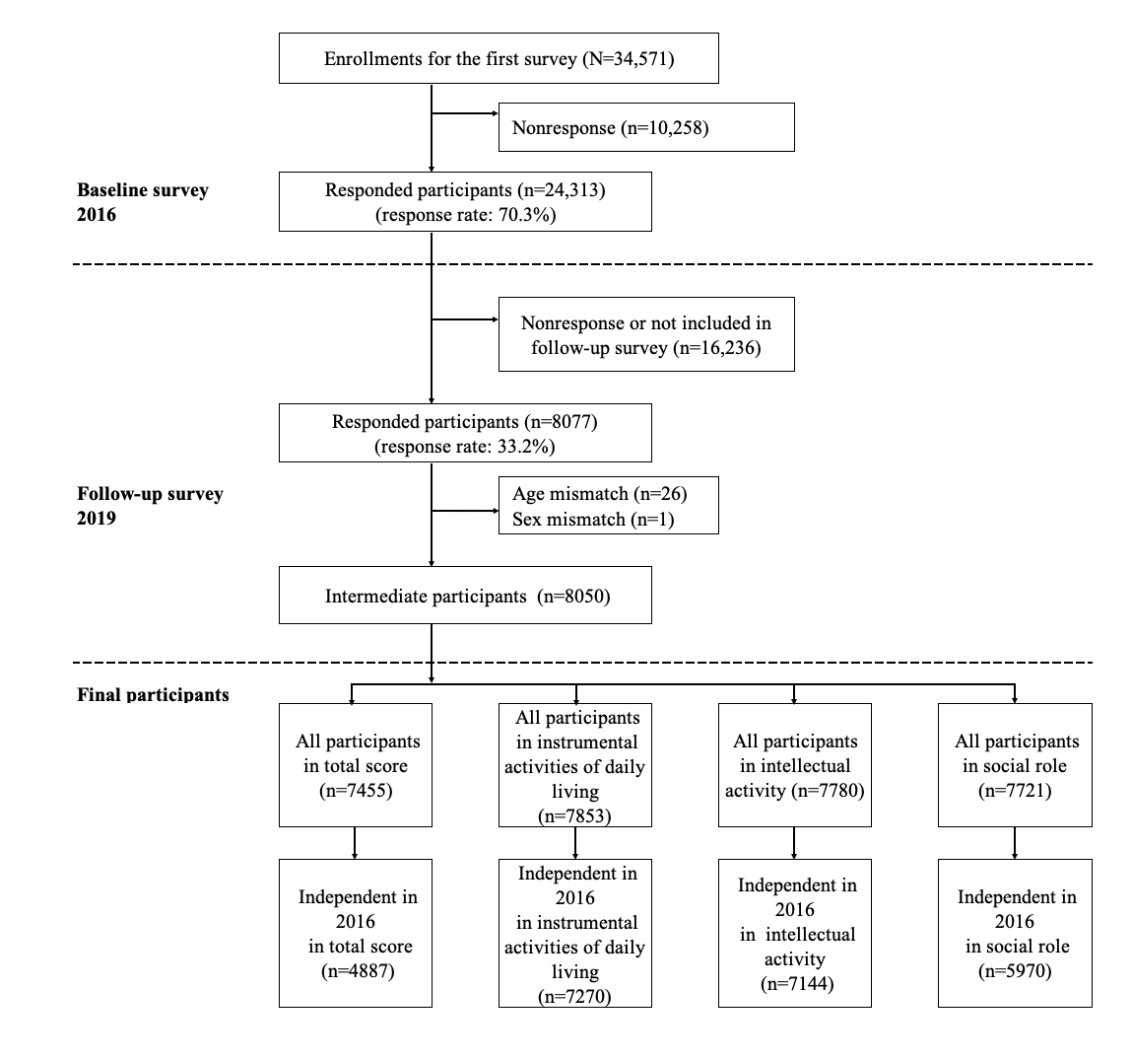
Participant recruitment process for the study.

Thus, the study focused on individuals who maintained their independence in 2016. Participants were selected based on their independent HLFC (total score of 3 subscales, namely, IADL, intellectual activity, and social role) in 2016. Those who did not meet the criteria for independent HLFC in 2016 were excluded, resulting in a final analytical sample of 4887 individuals in total score, 7270 in IADL, 7144 in intellectual activity, and 5970 in social role. A flowchart detailing the participant selection process is provided in Figure 1.

### Ethical Considerations

This study was approved by the Ethics Committee of the National Center for Geriatrics and Gerontology (No. 992, 1274-2), the Ethics Committee of Chiba University (No. 2493, 3442), and Hamamatsu University School of Medicine (approval number: 91-123). Informed consent was obtained by requiring all respondents to select an acceptance checkbox on the questionnaire before returning it. The study used anonymized data. The participants were not compensated.

### Outcome Variable: HLFC

HLFC was assessed using the Tokyo Metropolitan Institute of Gerontology Index of Competence (TMIG-IC) [7], which is grounded in the Lawton Instrumental Activities of Daily Living Scale. This 13-item index comprises three subscales of competence, each requiring a yes or no response, which are (1) IADL (5 items: the capability to travel by train or bus, shop for daily necessities, cook, pay bills, and manage a bank and postal savings account); (2) intellectual activity (4 items: the capacity to complete paperwork, read newspapers, read books or magazines, and interest in health-related articles or television programs); and (3) social role (4 items: the capability to visit friends’ homes, offer advice to family members or friends, visit ill individuals, and initiate conversations with young people; Table S1 in Multimedia Appendix 1). The TMIG-IC is extensively used as an HLFC evaluation tool. Scores for each subscale were calculated by summing the responses (yes=1 and no=0), with a higher score indicating greater competence. The maximum scores for the total score, IADL, intellectual activity, and social role are 13, 5, 4, and 4 points, respectively. Based on previous research [42,43], we defined a decline in HLFC subscales as (1) a reduction of ≥2 points in the total score, (2) a reduction of ≥1 point in IADL, (3) a reduction of ≥2 points in intellectual activity, and (4) a reduction of ≥2 points in social role. Participants with a high total score (12 or 13 points), IADL (5 points), intellectual activity (3 or 4 points), and social role (3 or 4 points) were considered to have maintained integrity in HLFC subscales, respectively. In this study, intactness in subscales was classified as “independent,” and decline in subscales was classified as “dependent,” respectively.

### Explanatory Variables

#### Internet Use

The 2016 frequency of internet use served as the variable for internet use. The pertinent question was “Have you used the Internet or emails in the past year? If yes, please specify the frequency.” The response options were “1. no,” “2. yes (less than a few times a month),” “3. yes (two to three times a week),” and “4. yes (almost every day).” Those who reported yes (2, 3, and 4) were categorized as internet users, and the response “1. no” identified internet nonusers.

#### Educational Attainment

Educational attainment was gauged by the completed years of schooling (≤9, 10-12, and ≥13 years).

### Covariates

Covariates included sex, age (65-69, 70-74, 75-79, 80-84, and ≥85 years), annual household equivalized income (3 quantiles: low, middle, and high), employment status (never employed, retired or not employed, and employed), marital status (married, widowed, divorced, never-married, and other), living arrangement (living with someone or living alone), self-rated health (good or poor), BMI (<18.5, 18.5-25, 25-30, and >30 kg/m^2^), diseases under treatment (hypertension, diabetes mellitus, stroke, cardiac diseases, cancer, and respiratory diseases; no or yes) [9,44], depression (no or yes), and population density (metropolitan, urban, semiurban, and rural). The 15-item Geriatric Depression Scale was used, defining mild depression as >5 points and severe depression as >10 points [45,46]. For depression data, responses with up to 2 missing items were included, with missing values replaced by the mean of the answers of respondents to the items. Population density was calculated for each municipality by dividing the population by the habitable land area, resulting in the number of residents per km² per unit area. Municipalities were then categorized based on the population density of the habitable area into 4 groups, that are metropolitan (≥4000/km²), urban (1500-3999/km²), semiurban (1000-1499/km²), and rural (<1000/km²). Missing data for each variable were assigned to missing groups, except for sex, age, and population density.

### Statistical Analysis

A descriptive analysis delineated the baseline characteristics of the study participants, differentiating between internet users and nonusers, and assessed the correlations between internet use and HLFC, as well as educational attainment and HLFC in 2016. Items missing from the 2019 HLFC variable, along with missing and other items from the educational attainment variable, were excluded from the analysis. HLFC changes in 2019 were set as binary outcomes, some showing a common prevalence (>10%). Therefore, we used Poisson regression analysis, adjusted for robust SE, and computed the risk ratio (RR) and 95% CI for the decline in HLFC in 2019 while avoiding overestimation [47]. To examine the impact of educational attainment on the association between internet use and HLFC decline, we conducted stratified analyses by educational level using a single model with all covariates included simultaneously. Significance tests were performed for each main effect and interaction, with statistical significance set at *P*<.05. Statistical analyses were carried out using Stata SE/15.0 (Stata Corp).

## Results

### Characteristics of Participants

The baseline characteristics of internet users and nonusers in 2016 are presented in Table 1. While no significant gender disparity in internet use was observed (*P*=.01), a strong correlation between educational attainment and income with internet use was noted (both *P*<.001). In addition, an inverse relationship between age and internet use was identified. The internet use rate was significantly higher among those who were currently working or had worked in the past compared with those who had never been employed (*P*<.001). Similarly, individuals residing in metropolitan and urban areas demonstrated a significantly higher internet use rate than those in rural areas (*P*<.001). Cross-tables that compare internet use or educational attainment with HLFC are depicted in Tables 2 and 3 and Table S2 in Multimedia Appendix 1. Older adults who used the internet showed higher independence across all subscales, including the total score, IADL, intellectual activity, and social role, compared with those who did not use the internet (Table 2). The lower the educational attainment, the fewer the number of older adults who exhibited independence in the total score and all subscales (Table 3).

**Table 1:** Characteristics of study participants by internet use in 2016 (n=8050). Those with missing values for internet use in 2016 were omitted (n=524).

Variables			Internet use (2016)				*P* value
			Yes (n=4336)	No (n=3190)			
**Sex, n (%)**							.01
	Male	2116 (55.9)		1452 (38.4)			
	Female	2220 (52)		1738 (40.7)			
**Age (years), n (%)**							<.001
	65-69	1824 (69.9)		672 (25.8)			
	70-74	1357 (58.1)		834 (35.7)			
	75-79	771 (41.7)		928 (50.2)			
	80-84	318 (33.9)		541 (57.7)			
	≥85	66 (21)		215 (67.2)			
**Income (3 quantiles), n (%)**							<.001
	Q1 (low)	990 (42.4)		1189 (50.9)			
	Q2 (middle)	1353 (61.9)		726 (33.2)			
	Q3 (high)	1441 (69.2)		552 (26.5)			
	Missing	552 (38.2)		723 (50)			
**Educational attainment (years), n (%)**							<.001
	≤9	631 (29.8)		1288 (60.7)			
	10-12	1956 (56.6)		1298 (37.6)			
	≥13	1721 (72.3)		559 (23.5)			
	Other	11 (37)		16 (53)			
	Missing	17 (27)		29 (47)			
**Employment status, n (%)**							<.001
	Never employed	171 (38.4)		244 (54.8)			
	Retired or not employed	2430 (56.6)		1643 (38.3)			
	Employed	1270 (60.7)		705 (33.7)			
	Missing	465 (38.1)		598 (49)			
**Marital status, n (%)**							<.001
	Married	3409 (57.1)		2227 (37.3)			
	Widowed	596 (43.6)		651 (47.7)			
	Divorced	177 (55)		126 (39.1)			
	Never married	116 (45.7)		119 (46.9)			
	Other	22 (37)		26 (44)			
	Missing	16 (22)		41 (55)			
**Living arrangement, n (%)**							<.001
	Living with someone	3610 (55.5)		2500 (38.5)			
	Living alone	580 (49.1)		515 (43.6)			
	Missing	146 (39.9)		175 (47.8)			
**Self-rated health, n (%)**							<.001
	Good	3902 (55.7)		2661 (38)			
	Poor	353 (41.3)		439 (51.4)			
	Missing	81 (42)		90 (46)			
**BMI (kg/m²), n (%)**							<.001
	<18.5	255 (50)		220 (43.1)			
	18.5-25	3161 (55.6)		2174 (38.3)			
	25-30	803 (51.6)		639 (41)			
	>30	80 (49)		73 (45)			
	Missing	37 (27)		84 (60)			
**Diseases under treatment, n (%)**							.003
	No	1826 (56.2)		1232 (37.9)			
	Yes	2319 (52.6)		1801 (40.8)			
	Missing	191 (49.6)		157 (40.5)			
**Depression, n (%)**							<.001
	No	3541 (57)		2311 (37.2)			
	Yes	631 (42.9)		732 (49.7)			
	Missing	164 (45.1)		147 (40.4)			
**Population density, n (%)**							<.001
	Metropolitan	2184 (61.6)		1196 (33.7)			
	Urban	1286 (54.7)		910 (38.7)			
	Semiurban	503 (48.6)		457 (44.1)			
	Rural	363 (32.4)		627 (56)			

**Table 2:** Higher-level functional capacity of independent and dependent individuals by internet use in 2016. Higher-level functional capacity comprises 3 subscales: IADL^a^, intellectual activity, and social role. The total scores on the 3 subscales were also calculated.

			2016 Internet use					*P* value
			Yes, n (%)	No, n (%)	Missing, n (%)			
**Total score^b^ (n=7455)**								<.001
	Independent^c^ (n=4887)	2953 (68.1)		1644 (51.5)	290 (55.3)			
	Dependent^d^ (n=2568)	1172 (27)		1235 (38.7)	161 (30.7)			
**IADL (n=7853)**								<.001
	Independent (n=7270)	4063 (93.7)		2749 (86.2)	458 (87.4)			
	Dependent (n=583)	201 (4.6)		340 (10.7)	42 (8)			
**Intellectual activity** **(n** **=7780)**								<.001
	Independent (n=7144)	4019 (92.7)		2683 (84.1)	442 (84.4)			
	Dependent (n=636)	225 (5.2)		367 (11.5)	44 (8)			
**Social role** **(n** **=7721)**								<.001
	Independent (n=5970)	3459 (79.8)		2147 (67.3)	364 (69.5)			
	Dependent (n=1751)	752 (17.3)		884 (27.7)	115 (21.9)			

^a^IADL: instrumental activities of daily living.

^b^Missing values were omitted for the total score and 3 subscales.

^c^Cutoff values for “independent” in the Tokyo Metropolitan Institute of Gerontology Index of Competence (TMIG-IC): “total score 12-13/13 points,” “IADL 5/5 points,” “intellectual activity 3-4/4 points,” and “social role 3-4/4 points” [42,43].

^d^Cutoff values for “dependent” in the TMIG-IC: “total score 1-11/13 points,” “IADL 1-4/5 points,” “intellectual activity 1-2/4 points,” and “social role 1-2/4 points.”

**Table 3:** Higher-level functional capacity of independent and dependent individuals by educational attainment in 2016. Higher-level functional capacity comprises 3 subscales: IADL^a^, intellectual activity, and social role. The total scores on the 3 subscales were also calculated.

		2016 Educational attainment (years)						*P* value	
		≤9, n (%)	10-12, n (%)	≥13, n (%)	Other, n (%)	Missing, n (%)			
**Total score^b^ (n=7455)**									<.001
	Independent^c^ (n=4887)	1081 (51)	2206 (63.8)	1559 (65.5)	14 (47)	27 (44)			
	Dependent^d^ (n=2568)	818 (38.6)	1005 (29.1)	714 (30)	12 (40)	19 (31)			
**IADL** **(n** **=7853)**									<.001
	Independent (n=7270)	1838 (86.7)	3172 (91.8)	2188 (91.9)	25 (83)	47 (76)			
	Dependent (n=583)	210 (9.9)	207 (6)	154 (6.5)	4 (13)	8 (13)			
**Intellectual activity** **(n** **=7780)**									<.001
	Independent (n=7144)	1734 (81.8)	3129 (90.5)	2215 (93)	23 (77)	43 (69)			
	Dependent (n=636)	282 (13.3)	218 (6.3)	124 (5.2)	4 (13)	8 (13)			
**Social role** **(n** **=7721)**									<.001
	Independent (n=5970)	1481(69.8)	2620 (75.8)	1812 (76.1)	20 (67)	37 (60)			
	Dependent (n=1751)	530 (25)	700 (20.3)	500 (21)	7 (23)	14 (23)			

^a^IADL: instrumental activities of daily living.

^b^Missing values were omitted for the total score and 3 subscales.

^c^Cutoff values for “independent” in Tokyo Metropolitan Institute of Gerontology Index of Competence (TMIG-IC): “total score 12-13/13 points,” “IADL 5/5 points,” “intellectual activity 3-4/4 points,” and “social role 3-4/4 points” [42,43].

^d^Cutoff values for “dependent” in TMIG-IC: “total score 1-11/13 points,” “IADL 1-4/5 points,” “intellectual activity 1-2/4 points,” and “social role 1-2/4 points.”

Moreover, among all subscales, the proportion of older adults independent in social roles was the lowest. In addition, the relationships exhibited similar trends across responses to the TMIG-IC’s 13 questions (Table S3 in Multimedia Appendix 1). The baseline characteristics of the participants by educational attainment in 2016 indicated an inverse relationship between age and educational attainment (Table S4 in Multimedia Appendix 1).

### Results of the Poisson Regression Analysis

Upon categorizing the independent individuals of each subgroup by 3 levels of educational attainment in 2016, we analyzed their internet use. The results revealed that internet users exhibited a suppressed decline in independence across the total score, IADL, intellectual activity, and social role subscales in 2019. Notably, among older adults with lower levels of education, significant suppression effects were observed in the total score and all subscales (Table 4).

**Table 4:** Associations between internet use in 2016 and suppression of higher-level functional capacity (HLFC) decline in 2019, segmented by educational attainment. In 2016, study participants were older adults with “independent” total score, IADL^a^, intellectual activity, and social role. Items missing in the 2019 HLFC variable were excluded from the verification targets.

Educational attainment (years) and internet use		HLFC									
		Total score		IADL			Intellectual activity			Social role	
		RR^b,c^ (95% CI)	*P* value	RR (95% CI)	*P* value	RR (95% CI)		*P* value	RR (95% CI)		*P* value
**≤9^d^ (total score: n=928; IADL: n=1757; intellectual activity: n=1619; social role: n=1353)**											
	No	Reference	—^e^	Reference	—	Reference		—	Reference		—
	Yes	0.57 (0.43-0.76)	<.001	0.58 (0.38-0.91)	.02	0.60 (0.41-0.89)		.01	0.74 (0.56-0.97)		.03
	Missing	0.95 (0.68-1.32)	.75	0.87 (0.52-1.46)	.60	0.76 (0.47-1.22)		.25	0.68 (0.47-0.98)		.04
**10**-**12 (total score: n=2077; IADL: n=3095; intellectual activity: n=3018; social role: n=2531)**											
	No	Reference	—	Reference	—	Reference		—	Reference		—
	Yes	0.78 (0.63-0.98)	.03	0.59 (0.39-0.90)	.01	0.91 (0.63-1.31)		.61	0.82 (0.68-1.00)		.05
	Missing	0.83 (0.59-1.17)	.28	0.71 (0.34-1.52)	.38	0.29 (0.07-1.24)		.10	0.71 (0.48-1.05)		.09
≥**13 (total score: n=1486; IADL: n=2141; intellectual activity: n=2163; social role: n=1755)**											
	No	Reference	—	Reference	—	Reference		—	Reference		—
	Yes	0.65 (0.51-0.85)	.001	0.55 (0.36-0.83)	.01	0.64 (0.37-1.10)		.11	0.83 (0.64-1.08)		.17
	Missing	0.89 (0.50-1.58)	.69	0.62 (0.28-1.37)	.24	0.95 (0.34-2.65)		.93	1.15 (0.59-2.21)		.68

^a^IADL: instrumental activities of daily living.

^b^RR: risk ratio.

^c^Risk ratio with adjustment for sex, age, annual household equivalized income, employment status, marital status, living arrangement, self-rated health, BMI, diseases under treatment, depression, and population density.

^d^Both missing and other items from the educational attainment variable were excluded from the verification targets.

^e^Not applicable.

Compared with nonusers, internet users with ≤9 years of educational attainment experienced a reduced decline in the total score in 2019 (RR 0.57, 95% CI 0.43-0.76; *P*<.001) and in the subscales of IADL (RR 0.58, 95% CI 0.38-0.91; *P*=.02), intellectual activity (RR 0.60, 95% CI 0.41-0.89; *P*=.01), and social role (RR 0.74, 95% CI 0.56-0.97; *P=*.03). Similarly, those in the 10-12 years of educational attainment group showed a mitigated decline in the total score (RR 0.78, 95% CI 0.63-0.98; *P=*.03), IADL (RR 0.59, 95% CI 0.39-0.90; *P*=.01), intellectual activity (RR 0.91, 95% CI 0.63-1.31; *P=*.61), and social role (RR 0.82, 95% CI 0.68-1.00; *P*=.05). The group with ≥13 years of educational attainment demonstrated a lesser decline in the total score (RR 0.65, 95% CI 0.51-0.85; *P*=.001), IADL (RR 0.55, 95% CI 0.36-0.83; *P*=.01), intellectual activity (RR 0.64, 95% CI 0.37-1.10; *P=*.11), and social role (RR 0.83, 95% CI 0.64-1.08; *P=*.17; Table 4). No interaction was identified between internet use and educational attainment (Table S5 in Multimedia Appendix 1).

## Discussion

### Principal Findings

This study explored the relationship between internet use and the maintenance of independence in HLFC among older adults, including the varying impacts based on educational attainment, through analysis of panel data from longitudinal surveys in 2016 and 2019. In this extensive population-based analysis, internet use among older adults was significantly linked to the suppression of HLFC decline, irrespective of educational level. Specifically, significant effects in mitigating decline were notable in all HLFC subscales, including IADL, intellectual activity, social role, and the total score, particularly in older adults with low education (≤9 years). These findings indicate that older adults with lower levels of education could benefit from internet use in suppressing HLFC decline. To our knowledge, this is the inaugural study to assess the relationship between internet use and the mitigation of HLFC decline among older adults with low education, by segmenting based on their educational attainment, an aspect challenging to enhance retrospectively.

### HLFC Can Contribute to the Independent Living of Older Adults

Many older adults prefer to remain in their familiar residences [2]. Hence, maintaining a relatively independent HLFC, which encompasses capabilities, such as using public transportation, shopping, cooking, accessing information from various sources, and socializing with family, friends, and acquaintances, is crucial for fostering independent living. However, functional capacity tends to diminish with age [6], posing a risk of encountering numerous health challenges [9,10]. Consequently, preserving and enhancing HLFC plays a pivotal role in promoting the health of older adults and potentially extending their capability to live independently within their communities.

### HLFC Could Benefit from Improved IADL Through Internet Use

A digital divide among older adults has been linked to educational disparities [40]. Moreover, a survey in Japan highlighted a digital divide wherein internet use among older adults is increasing, while the proportion of lower-income individuals using the internet remains low [16]. This study corroborated that older adults with lower education or income levels were less likely to be internet users compared with those with higher education or income levels. However, numerous studies have documented the health improvement effects of ICT use, including the internet, irrespective of educational attainment or income. Our findings align with previous research, indicating that internet and email use can likely mitigate and improve the risk of IADL decline [34,35]. IADL impairment may signal early reductions in physical and cognitive functions, potentially leading to adverse health outcomes, increased incidents of heart failure, and mortality among older adults [48]. Factors associated with IADL include social isolation [49], depression [50,51], subjective health [50,52], cognitive function [52], intellectual activity [53-55], social role [54,55], community-level social capital [56], and social participation [57,58]. The health improvement effects gained through internet use, such as mitigating social isolation and loneliness [24,27,29,33,35-39], preventing and reducing depression [21-23,32,33], maintaining good subjective health [23,33,35], reducing dementia risk [19], enhancing cognitive function [18,20,31], and fostering social participation [28,39], overlap with factors related to IADL. Our results suggest that internet use may aid in suppressing HLFC decline by enhancing factors associated with IADL, a key component of HLFC.

The benefits of internet use are interrelated. For instance, internet users exhibit lower levels of social isolation compared with nonusers [37], and ICT use has been shown to reduce social isolation among older adults through 4 mechanisms: connecting with the external world, acquiring social support, pursuing activities of interest, and enhancing self-confidence [27]. In addition, while nonusers demonstrated a decline in social contact, internet users maintained stable social connections and alleviated loneliness [36], consistent with findings that social contact and perceived social support mediate the relationship between social media communication and reduced loneliness levels [38]. Internet use alleviates loneliness [24,29,33,35,36,38,39] and contributes to improved self-rated health, reduced chronic illnesses, increased subjective well-being, and fewer depressive symptoms through the mediated effect of decreased loneliness [33]. Beyond preventing and ameliorating depression [21-23,32,33] and enhancing self-rated health [23,33,35], positive effects on cognitive function [18,20,31] have also been reported. Moreover, beneficial impacts on well-being [25,26,33,35,39], quality of life [22], and health literacy [30] have been confirmed. Thus, the influence of internet use on the daily lives of older adults may be substantial.

Many older adults in Japan use the internet to connect with family, friends, and acquaintances and to discover interesting information [16]. Given that internet use constitutes an “intellectual activity” in itself, using it for instrumental, informational, and social purposes [39] may further mitigate the decline in “intellectual activity” activities, such as “completing paperwork,” “reading newspapers,” “reading books or magazines,” and “showing interest in health-related articles or television programs.”

Increased social participation [28] and diverse volunteer activities [39] through internet use could establish a foundation for exchange relationships that encourage actions, such as “visiting friends’ homes,” “offering advice to family members or friends,” “visiting ill individuals,” and “initiating conversations with young people,” thereby preventing the decline in social roles. Strong associations between good intellectual activity and social role with remaining independent in IADL [53,54], along with reports that impairments in social role and intellectual activity not only often precede IADL disability but also significantly forecast the onset of IADL disability [55], suggest that enhancements in “intellectual activity” and “social role” through internet use could lead to improved IADL and, subsequently, help suppress the overall decline in HLFC.

### Internet Use May Mitigate Educational Inequality in HLFC

Socioeconomic disparities were evident across all subscales of HLFC, underscoring educational inequalities [6] (Table 3 and Table S2 in Multimedia Appendix 1). Consequently, to address health disparities among older adults in the community, it is imperative to implement strategies to preserve the HLFC of older adults, especially those with lower levels of education, although enhancing educational attainment retrospectively poses a significant challenge.

This study found that even at baseline, older adults who used the internet tended to maintain higher levels of independence in terms of the total score, IADL, intellectual activity, and social role compared with nonusers. Furthermore, longitudinal analysis revealed that internet use significantly curbed the decline in HLFC among older adults, irrespective of their educational background.

Recognized for fostering social participation among older adults [28], internet use may counteract the trend where wealthier or more educated older adults are more engaged in social activities [59]. Social participation can diminish the risk of loneliness across socioeconomic statuses, potentially serving as a countermeasure to health disparities [60]. Moreover, community activity participation has been identified as a key mediator in the relationship between educational level and incident functional disability among older adults aged 65-74 years [61], suggesting that enhanced social participation through internet use [28] may mediate the link between educational inequality and HLFC in this study. In addition, internet use may serve as a proxy for continuous educational attainment, with its protective effects independent of “past” education levels; instead, these effects may rely on middle- and late-life cognitive activity [19]. Late-life cognitive activity has been shown to impact subsequent cognitive health [62], suggesting that this study may illustrate how internet use later in life, as a form of continuous educational attainment, could improve educational inequality in HLFC.

### Limitations

This study has several limitations. First, since internet use was assessed in 2016, changes in use by 2019 were not captured. However, even previous internet use experiences may have contributed to suppressing HLFC decline in older adults, warranting further investigation into the impact of internet use duration. Second, the study did not account for the purpose of internet use. The analysis encompassed a broad range of internet activities, including communication, health and medical information search, navigation, online shopping, banking, and stock trading. These activities represent the majority of internet use by older adults, suggesting the study’s findings broadly reflect the positive impact of diverse internet use on maintaining HLFC independence. Third, the validity and reliability of the TMIG-IC as an evaluative tool for HLFC were not addressed. With the changing lifestyles of older adults, there is a call for developing scales that can measure higher levels of HLFC reflective of these changes. In addition, considering the psychological aspects of IADL, future studies might benefit from using scales with psychometric properties. Fourth, our data included only 2 waves. We acknowledge that interpreting results from only 2 time points requires caution because longitudinal data with 3 or more time points would provide a stronger basis for capturing dynamic links between exposures and outcomes [63]. Finally, using data from the JAGES study, comprising healthy participants, might have led to overestimating the association between internet use and HLFC independence.

### Conclusion

In conclusion, this study indicates that internet use can support the maintenance of HLFC independence among older adults with higher education and those with lower education levels. Internet use can mitigate declines in key components of HLFC, such as IADL, intellectual activity, and social role, which are essential for independent living in familiar communities, even for older adults with low educational attainment. These findings underscore the importance of promoting internet use among older adults to support their independence. Moreover, engaging with more devices and applications is linked to fewer functional limitations, higher life satisfaction, and greater goal attainment [35]. Thus, expanding internet access and use among older adults, particularly those with lower education, is vital for reducing functional health disparities attributed to educational attainment.

## Appendix

Multimedia Appendix 1 The Tokyo Metropolitan Institute of Gerontology Index of Competence (TMIG-IC); characteristics of the study participants, by higher-level functional capacity (HLFC) and each independence; answer results of TMIG-IC by educational attainment; characteristics of study participants by educational attainment in 2016; and interaction.

## Abbreviations

HLFC: higher-level functional capacity
IADL: instrumental activities of daily living
ICT: information and communication technology
JAGES: Japan Gerontological Evaluation Study
RR: risk ratio
TMIG-IC: Tokyo Metropolitan Institute of Gerontology Index of Competence
