# Supplementary material for: Internet Use and Higher-Level Functional Capacity Decline Suppression in Japanese Older Adults With Low Education: JAGES 2016-2019 Longitudinal Study
Source: JMIR Aging. 2024 Sep 20;7:e53384. doi: 10.2196/53384 (PMC11452757; doi:10.2196/53384)
Supplement: Multimedia Appendix 1 [file aging_v7i1e53384_app1.docx]

**Multimedia Appendix 1**

**Table S1.** The Tokyo Metropolitan Institute of Gerontology Index of Competence (TMIG-IC).

**Table S2.** Characteristics of the study participants, by Higher-Level Functional Capacity (HLFC) and each independence.

**Table S3.** Answer Results of TMIG-IC by educational attainment.

**Table S4.** Characteristics of study participants by educational attainment in 2016.

**Table S5.** Interaction

Table S1: The Tokyo Metropolitan Institute of Gerontology Index of Competence (TMIG-IC)

| IADL (5) | 1) Can you go out alone by train or bus? |
| --- | --- |
|  | 2) Can you go shopping for daily necessities? |
|  | 3) Can you cook for yourself? |
|  | 4) Can you pay your bills by yourself? |
|  | 5) Can you deposit or withdraw money from your bank / postal savings  account(s) by yourself? |
|  |  |
| Intellectual activity (4) | 6) Can you complete the paperwork for your pension, etc. by yourself? |
|  | 7) Do you read newspapers? |
|  | 8) Do you read books or magazines? |
|  | 9) Are you interested in health-related articles or television programs? |
|  |  |
| Social role (4) | 10) Do you visit your friends’ homes? |
|  | 11) Do you give advice to your family members or friends? |
|  | 12) Can you visit people who have fallen ill? |
|  | 13) Do you start conversations with young people? |

Table S2-1: Characteristics of the study participants, by Higher-Level Functional Capacity (HLFC) and each independence.

[Total score and IADL]

|  |  | Total score n=7455 | | |  | IADL n=7853  | | |
| --- | --- | --- | --- | --- | --- | --- | --- | --- |
|  |  | Independent (4887) | Dependent (2568) |  |  | Independent (7270) | Dependent (583) |  |
| Variables in 2016 |  | n (%) | n (%) | *p* value |  | n (%) | n (%) | *p* value |
| Internet use | Yes | 2953 (68.1) | 1172 (27.0) | <.001 |  | 4063 (93.7) | 201 (4.6) | <.001 |
|  | No | 1644 (51.5) | 1235 (38.7) |  |  | 2749 (86.2) | 340 (10.7) |  |
|  | Missing | 290 (55.3) | 161 (30.7) |  |  | 458 (87.4) | 42 (8) |  |
| Sex | Male | 1979 (52.3) | 1563 (41.3) |  |  | 3227 (85.3) | 467 (12.3) |  |
|  | Female | 2908 (68.2) | 1005 (23.6) |  |  | 4043 (94.8) | 116 (2.7) |  |
| Age (years) | 65-69 | 1621 (62.2) | 853 (32.7) |  |  | 2409 (92.4) | 149 (5.7) |  |
|  | 70-74 | 1489 (63.8) | 699 (30.0) |  |  | 2127 (91.1) | 160 (6.9) |  |
|  | 75-79 | 1145 (61.9) | 553 (29.9) |  |  | 1662 (89.8) | 143 (7.7) |  |
|  | 80-84 | 483 (51.5) | 331 (35.3) |  |  | 814 (86.8) | 81 (9) |  |
|  | 85 years and older | 149 (46.6) | 132 (41.3) |  |  | 258 (80.6) | 50 (16) |  |
| Income | Q1 (low) | 1251 (53.6) | 897 (38.4) |  |  | 2079 (89.0) | 192 (8.2) |  |
| (3 quantiles) | Q2 (middle) | 1394 (63.8) | 666 (30.5) |  |  | 1991 (91.1) | 151 (6.9) |  |
|  | Q3 (high) | 1416 (68.0) | 570 (27.4) |  |  | 1901 (91.2) | 151 (7.3) |  |
|  | Missing | 826 (57.2) | 435 (30.1) |  |  | 1299 (89.9) | 89 (6) |  |
| Educational attainment | ≤ 9 | 1081 (51.0) | 818 (38.6) |  |  | 1838 (86.7) | 210 (9.9) |  |
| (years) | 10–12 | 2206 (63.8) | 1005 (29.1) |  |  | 3172 (91.8) | 207 (6.0) |  |
|  | ≥ 13 | 1559 (65.5) | 714 (30.0) |  |  | 2188 (91.9) | 154 (6.5) |  |
|  | Other | 14 (47) | 12 (40) |  |  | 25 (83) | 4 (13) |  |
|  | Missing | 27 (44) | 19 (31) |  |  | 47 (76) | 8 (13) |  |
| Employment status | Never employed | 255 (57.3) | 152 (34.2) |  |  | 400 (89.9) | 34 (8) |  |
|  | Retired/Not employed | 2605 (60.7) | 1433 (33.4) |  |  | 3911 (91.1) | 307 (7.2) |  |
|  | Employed | 1342 (64.2) | 619 (29.6) |  |  | 1880 (89.9) | 158 (7.6) |  |
|  | Missing | 685 (56.1) | 364 (29.8) |  |  | 1079 (88.4) | 84 (7) |  |
| Marital status | Married | 3682 (61.6) | 1906 (31.9) |  |  | 5352 (89.6) | 495 (8.3) |  |
|  | Widowed | 845 (61.9) | 385 (28.2) |  |  | 1259 (92.2) | 70 (5) |  |
|  | Divorced | 177 (55.0) | 118 (36.7) |  |  | 308 (95.7) | 6 (2) |  |
|  | Never-married | 127 (50.0) | 113 (44.5) |  |  | 241 (94.9) | 7 (3) |  |
|  | Other | 26 (44) | 24 (41) |  |  | 53 (90) | 0 (0) |  |
|  | Missing | 30 (41) | 22 (30) |  |  | 57 (77) | 5 (7) |  |
| Living  arrangement | Living with someone | 4000 (61.5) | 2064 (31.7) |  |  | 5818 (89.5) | 538 (8.3) |  |
|  | Living alone | 683 (57.8) | 391 (33.1) |  |  | 1133 (95.9) | 20 (2) |  |
|  | Missing | 204 (55.7) | 113 (30.9) |  |  | 319 (87.2) | 25 (7) |  |
| Self–rated health | Good | 4435 (63.4) | 2088 (29.8) |  |  | 6400 (91.4) | 454 (6.5) |  |
|  | Poor | 345 (40.4) | 429 (50.2) |  |  | 708 (82.8) | 116 (13.6) |  |
|  | Missing | 107 (54.9) | 51 (26) |  |  | 162 (83.1) | 13 (7) |  |
| BMI | < 18.5 | 295 (57.8) | 179 (35.1) |  |  | 468 (91.8) | 35 (7) |  |
| (Kg/m²) | 18.5-25 | 3501 (61.6) | 1786 (31.4) |  |  | 5154 (90.7) | 399 (7.0) |  |
|  | 25-30 | 933 (59.9) | 502 (32.2) |  |  | 1384 (88.9) | 128 (8.2) |  |
|  | > 30 | 98 (61) | 54 (33) |  |  | 145 (89.5) | 13 (8) |  |
|  | Missing | 60 (43) | 47 (34) |  |  | 119 (85.6) | 8 (6) |  |
| Diseases  under treatment | No | 2059 (63.3) | 988 (30.4) |  |  | 2983 (91.7) | 203 (6.2) | .002 |
|  | Yes | 2588 (58.7) | 1478 (33.5) |  |  | 3943 (89.4) | 356 (8.1) |  |
|  | Missing | 240 (62.3) | 102 (26.2) |  |  | 344 (89.1) | 24 (6) |  |
| Depression | No | 4141 (66.6) | 1689 (27.2) |  |  | 5682 (91.4) | 399 (6.4) |  |
|  | Yes | 574 (39.0) | 772 (52.5) |  |  | 1276 (86.7) | 162 (11.0) |  |
|  | Missing | 172 (47.3) | 107 (29.4) |  |  | 312 (85.7) | 22 (6) |  |
| Population density | Metropolitan | 2077 (58.6) | 1232 (34.8) |  |  | 3237 (91.3) | 232 (6.5) | .01 |
|  | Urban | 1438 (61.2) | 762 (32.4) |  |  | 2122 (90.3) | 180 (7.7) |  |
|  | Semiurban | 678 (65.4) | 271 (26.2) |  |  | 928 (89.6) | 76 (7) |  |
|  | Rural | 694 (62.0) | 303 (27.1) |  |  | 983 (87.9) | 95 (9) |  |

Table S2-2: Characteristics of the study participants, by Higher-Level Functional Capacity (HLFC) and each independence.

[Intellectual activity and Social role]

|  |  | Intellectual activity n=7780 | | |  | Social role n=7721 | | |
| --- | --- | --- | --- | --- | --- | --- | --- | --- |
|  |  | Independent (7144) | Dependent (636) |  |  | Independent (5970) | Dependent (1751) |  |
| Variables in 2016 |  | n (%) | n (%) | *p* value |  | n (%) | n (%) | *p* value |
| Internet use | Yes | 4019 (92.7) | 225 (5.2) | <.001 |  | 3459 (79.8) | 752 (17.3) | <.001 |
|  | No | 2683 (84.1) | 367 (11.5) |  |  | 2147 (67.3) | 884 (27.7) |  |
|  | Missing | 442 (84.4) | 44 (8) |  |  | 364 (69.5) | 115 (21.9) |  |
| Sex | Male | 3346 (88.4) | 336 (8.9) |  |  | 2554 (67.5) | 1107 (29.3) |  |
|  | Female | 3798 (89.0) | 300 (7.0) |  |  | 3416 (80.1) | 644 (15.1) |  |
| Age (years) | 65-69 | 2326 (89.2) | 231 (8.9) |  |  | 1953 (74.9) | 566 (21.7) |  |
|  | 70-74 | 2093 (89.7) | 168 (7.2) |  |  | 1802 (77.2) | 456 (19.5) |  |
|  | 75-79 | 1654 (89.4) | 129 (7.0) |  |  | 1399 (75.6) | 370 (20.0) |  |
|  | 80-84 | 804 (85.7) | 73 (8) |  |  | 626 (66.7) | 253 (27.0) |  |
|  | 85 years and older | 267 (83.4) | 35 (11) |  |  | 190 (59.4) | 106 (33.1) |  |
| Income | Q1 (low) | 2003 (85.8) | 259 (11.1) |  |  | 1611 (69.0) | 622 (26.6) |  |
| (3 quantiles) | Q2 (middle) | 2007 (91.8) | 122 (5.6) |  |  | 1640 (75.0) | 475 (21.7) |  |
|  | Q3 (high) | 1917 (92.0) | 119 (5.7) |  |  | 1670 (80.1) | 358 (17.2) |  |
|  | Missing | 1217 (84.2) | 136 (9.4) |  |  | 1049 (72.6) | 296 (20.5) |  |
| Educational attainment | ≤ 9 | 1734 (81.8) | 282 (13.3) |  |  | 1481 (69.8) | 530 (25.0) |  |
| (years) | 10–12 | 3129 (90.5) | 218 (6.3) |  |  | 2620 (75.8) | 700 (20.3) |  |
|  | ≥ 13 | 2215 (93.0) | 124 (5.2) |  |  | 1812 (76.1) | 500 (21.0) |  |
|  | Other | 23 (77) | 4 (13) |  |  | 20 (67) | 7 (23) |  |
|  | Missing | 43 (69) | 8 (13) |  |  | 37 (60) | 14 (23) |  |
| Employment status | Never employed | 373 (83.8) | 57 (13) |  |  | 314 (70.6) | 109 (24.5) |  |
|  | Retired/Not employed | 3875 (90.3) | 313 (7.3) |  |  | 3125 (72.8) | 1023 (23.8) |  |
|  | Employed | 1871 (89.4) | 160 (7.7) |  |  | 1644 (78.6) | 378 (18.1) |  |
|  | Missing | 1025 (84.0) | 106 (8.7) |  |  | 887 (72.7) | 241 (19.7) |  |
| Marital status | Married | 5379 (90.0) | 429 (7.2) |  |  | 4496 (75.3) | 1265 (21.2) |  |
|  | Widowed | 1183 (86.6) | 116 (8.5) |  |  | 1016 (74.4) | 269 (19.7) |  |
|  | Divorced | 263 (81.7) | 47 (15) |  |  | 220 (68.3) | 88 (27) |  |
|  | Never-married | 221 (87.0) | 26 (10) |  |  | 148 (58.3) | 98 (39) |  |
|  | Other | 47 (80) | 9 (15) |  |  | 43 (73) | 15 (25) |  |
|  | Missing | 51 (69) | 9 (12) |  |  | 47 (64) | 16 (22) |  |
| Living  arrangement | Living with someone | 5820 (89.5) | 490 (7.5) |  |  | 4882 (75.1) | 1378 (21.2) |  |
|  | Living alone | 1012 (85.6) | 111 (9.4) |  |  | 822 (69.5) | 300 (25.4) |  |
|  | Missing | 312 (85.3) | 35 (10) |  |  | 266 (72.7) | 73 (20) |  |
| Self–rated health | Good | 6301 (90.0) | 488 (7.0) |  |  | 5343 (76.3) | 1391 (19.9) |  |
|  | Poor | 676 (79.1) | 134 (15.7) |  |  | 485 (56.7) | 328 (38.4) |  |
|  | Missing | 167 (85.6) | 14 (7) |  |  | 142 (72.8) | 32 (16) |  |
| BMI | < 18.5 | 449 (88.0) | 45 (9) |  |  | 363 (71.2) | 128 (25.1) |  |
| (Kg/m²) | 18.5-25 | 5082 (89.4) | 425 (7.5) |  |  | 4241 (74.6) | 1220 (21.5) |  |
|  | 25-30 | 1368 (87.9) | 134 (8.6) |  |  | 1163 (74.7) | 333 (21.4) |  |
|  | > 30 | 144 (88.9) | 14 (9) |  |  | 119 (73.5) | 37 (23) |  |
|  | Missing | 101 (72.7) | 18 (13) |  |  | 84 (60) | 33 (24) |  |
| Diseases  under treatment | No | 2903 (89.3) | 257 (7.9) | .35 |  | 2471 (76.0) | 665 (20.5) |  |
|  | Yes | 3904 (88.5) | 349 (7.9) |  |  | 3208 (72.7) | 1021 (23.1) |  |
|  | Missing | 337 (87.3) | 30 (8) |  |  | 291 (75.6) | 65 (17) |  |
| Depression | No | 5648 (90.9) | 392 (6.3) |  |  | 4949 (79.6) | 1067 (17.2) |  |
|  | Yes | 1196 (81.3) | 219 (14.9) |  |  | 805 (54.7) | 594 (40.4) |  |
|  | Missing | 300 (82.4) | 25 (7) |  |  | 216 (59.3) | 90 (25) |  |
| Population density | Metropolitan | 3172 (89.5) | 279 (7.9) |  |  | 2522 (71.1) | 885 (25.0) |  |
|  | Urban | 2089 (88.9) | 187 (8.0) |  |  | 1766 (75.2) | 500 (21.3) |  |
|  | Semiurban | 941 (90.8) | 54 (5) |  |  | 804 (77.6) | 187 (18.1) |  |
|  | Rural | 942 (84.2) | 116 (10.4) |  |  | 878 (78.5) | 179 (16.0) |  |

Table S3: Answer Results of TMIG-IC by educational attainment

|  |  |  | 2016 | |  | 2019 | |  |
| --- | --- | --- | --- | --- | --- | --- | --- | --- |
| Subscale | Question | Education^a^ | YES | NO | *p* value | YES | NO | *p* value |
| **IADL** | travel by public transport | ≤ 9 | 2025 (97.1) | 61 (3) | <.001 | 1926 (93.3) | 138 (6.7) | <.001 |
|  |  | 10–12 | 3391 (99.2) | 26 (1) |  | 3349 (98.1) | 64 (2) |  |
|  |  | ≥ 13 | 2346 (99.5) | 13 (1) |  | 2309 (98.1) | 44 (2) |  |
|  |  |  |  |  |  |  |  |  |
|  | shop for daily necessities | ≤ 9 | 2053 (98.2) | 37 (2) | <.001 | 1981 (95.5) | 94 (5) | <.001 |
|  |  | 10–12 | 3399 (99.4) | 21 (1) |  | 3366 (98.8) | 41 (1) |  |
|  |  | ≥ 13 | 2348 (99.6) | 10 (0.4) |  | 2331 (98.9) | 26 (1) |  |
|  |  |  |  |  |  |  |  |  |
|  | cook | ≤ 9 | 1976 (94.4) | 117 (5.6) | .40 | 1933 (93.3) | 140 (6.8) | .004 |
|  |  | 10–12 | 3251 (95.1) | 167 (4.9) |  | 3251 (95.3) | 162 (4.8) |  |
|  |  | ≥ 13 | 2228 (94.5) | 131 (5.6) |  | 2209 (93.9) | 144 (6.1) |  |
|  |  |  |  |  |  |  |  |  |
|  | pay bills | ≤ 9 | 2036 (97.5) | 52 (3) | <.001 | 1982 (95.7) | 90 (4) | <.001 |
|  |  | 10–12 | 3397 (99.3) | 23 (1) |  | 3355 (98.4) | 53 (2) |  |
|  |  | ≥ 13 | 2344 (99.5) | 13 (1) |  | 2326 (98.8) | 28 (1) |  |
|  |  |  |  |  |  |  |  |  |
|  | manage a bank/postal savings account | ≤ 9 | 2012 (95.8) | 88 (4) | <.001 | 1958 (93.7) | 131 (6.3) | <.001 |
|  |  | 10–12 | 3385 (98.7) | 44 (1) |  | 3354 (98.0) | 67 (2) |  |
|  |  | ≥ 13 | 2344 (99.2) | 18 (1) |  | 2330 (98.7) | 30 (1) |  |
|  |  |  |  |  |  |  |  |  |
| **intellectual  activity** | complete paperwork | ≤ 9 | 1913 (91.8) | 171 (8.2) | <.001 | 1832 (88.6) | 237 (11.5) | <.001 |
|  |  | 10–12 | 3327 (97.4) | 90 (3) |  | 3288 (96.4) | 122 (3.6) |  |
|  |  | ≥ 13 | 2320 (98.3) | 41 (2) |  | 2279 (96.9) | 73 (3) |  |
|  |  |  |  |  |  |  |  |  |
|  | read newspapers | ≤ 9 | 1806 (87.0) | 269 (13.0) | <.001 | 1731 (84.3) | 322 (15.7) | <.001 |
|  |  | 10–12 | 3130 (91.7) | 282 (8.3) |  | 3040 (89.5) | 356 (10.5) |  |
|  |  | ≥ 13 | 2192 (93.1) | 163 (6.9) |  | 2126 (90.5) | 223 (9.5) |  |
|  |  |  |  |  |  |  |  |  |
|  | read books or magazines | ≤ 9 | 1448 (70.1) | 619 (30.0) | <.001 | 1345 (65.9) | 697 (34.1) | <.001 |
|  |  | 10–12 | 2848 (84.0) | 544 (16.0) |  | 2705 (79.9) | 681 (20.1) |  |
|  |  | ≥ 13 | 2108 (89.5) | 248 (10.5) |  | 2053 (87.4) | 296 (12.6) |  |
|  | interest in health-related articles or television programs | ≤ 9 | 1928 (92.6) | 155 (7.4) | .01 | 1864 (90.7) | 191 (9.3) | .01 |
|  |  | 10–12 | 3213 (94.1) | 202 (5.9) |  | 3166 (93.1) | 235 (6.9) |  |
|  |  | ≥ 13 | 2170 (92.0) | 188 (8.0) |  | 2168 (92.1) | 185 (7.9) |  |
|  |  |  |  |  |  |  |  |  |
| **Social role** | visit friends' homes | ≤ 9 | 1253 (60.4) | 821 (39.6) | .04 | 1119 (54.6) | 931 (45.4) | .05 |
|  |  | 10–12 | 2110 (61.9) | 1298 (38.1) |  | 1940 (57.2) | 1452 (42.8) |  |
|  |  | ≥ 13 | 1379 (58.5) | 977 (41.5) |  | 1269 (54.2) | 1072 (45.8) |  |
|  |  |  |  |  |  |  |  |  |
|  | offer advice to family members or friends | ≤ 9 | 1605 (77.3) | 472 (22.7) | <.001 | 1442 (70.7) | 599 (29.4) | <.001 |
|  |  | 10–12 | 2806 (82.7) | 587 (17.3) |  | 2675 (79.0) | 711 (21.0) |  |
|  |  | ≥ 13 | 1973 (83.8) | 381 (16.2) |  | 1860 (79.3) | 485 (20.7) |  |
|  |  |  |  |  |  |  |  |  |
|  | visit ill individuals | ≤ 9 | 1921 (92.8) | 150 (7.2) | <.001 | 1783 (87.8) | 247 (12.2) | <.001 |
|  |  | 10–12 | 3259 (95.7) | 147 (4.3) |  | 3176 (93.6) | 217 (6.4) |  |
|  |  | ≥ 13 | 2243 (95.7) | 102 (4.4) |  | 2187 (93.3) | 156 (6.7) |  |
|  |  |  |  |  |  |  |  |  |
|  | initiate conversations with  young people | ≤ 9 | 1606 (77.1) | 477 (22.9) | <.001 | 1457 (71.1) | 592 (28.9) | <.001 |
|  |  | 10–12 | 2775 (81.8) | 619 (18.2) |  | 2601 (77.0) | 777 (23.0) |  |
|  |  | ≥ 13 | 1907 (81.5) | 434 (18.5) |  | 1836 (78.3) | 508 (21.7) |  |

a Both missing and other items from the educational attainment variable were excluded from the verification targets.

Table S4: Characteristics of study participants by educational attainment in 2016

|  |  | educational attainment (2016) ^a^ | | | |
| --- | --- | --- | --- | --- | --- |
|  |  | ≤ 9 | 10–12 | ≥ 13 |  |
| 2016 |  | n (%) | n (%) | n (%) | *p* value |
| Sex | Male | 924 (24.4) | 1425 (37.7) | 1408 (37.2) | <.001 |
|  | Female | 1197 (28.1) | 2031 (47.6) | 973 (22.8) |  |
|  |  |  |  |  |  |
| Age (years) | 65-69 | 413 (15.8) | 1213 (46.5) | 958 (36.7) | <.001 |
|  | 70-74 | 594 (25.5) | 1047 (44.9) | 673 (28.8) |  |
|  | 75-79 | 594 (32.1) | 783 (42.3) | 454 (24.5) |  |
|  | 80-84 | 375 (40.0) | 312 (33.3) | 230 (24.5) |  |
|  | 85 years and older | 145 (45.3) | 101 (31.6) | 66 (21) |  |
|  |  |  |  |  |  |
| Income | Q1 (low) | 845 (36.2) | 981 (42.0) | 481 (20.6) | <.001 |
| (3 quantiles) | Q2 (middle) | 425 (19.4) | 1046 (47.9) | 707 (32.3) |  |
|  | Q3 (high) | 288 (13.8) | 903 (43.3) | 884 (42.4) |  |
|  | Missing | 563 (39.0) | 526 (36.4) | 309 (21.4) |  |
|  |  |  |  |  |  |
| Employment status | Never | 142 (31.9) | 183 (41.1) | 118 (26.5) | <.001 |
|  | Retired/Not employed | 1036 (24.1) | 1885 (43.9) | 1332 (31.0) |  |
|  | Employed | 487 (23.3) | 914 (43.7) | 673 (32.2) |  |
|  | Missing | 456 (37.4) | 474 (38.8) | 258 (21.1) |  |
|  |  |  |  |  |  |
| Marital status | Married | 1439 (24.1) | 2597 (43.5) | 1887 (31.6) | <.001 |
|  | Widowed | 460 (33.7) | 571 (41.8) | 314 (23.0) |  |
|  | Divorced | 88 (27) | 145 (45.0) | 84 (26) |  |
|  | Never-married | 79 (31) | 105 (41.3) | 67 (26) |  |
|  | Other | 20 (34) | 19 (32) | 16 (27) |  |
|  | Missing | 35 (47) | 19 (26) | 13 (18) |  |
|  |  |  |  |  |  |
| Living arrangement | Living with someone | 1645 (25.3) | 2792 (42.9) | 2000 (30.8) | <.001 |
|  | Living alone | 342 (28.9) | 510 (43.2) | 311 (26.3) |  |
|  | Missing | 134 (36.6) | 154 (42.1) | 70 (19) |  |
|  |  |  |  |  |  |
| Self–rated health | Good | 1765 (25.2) | 3043 (43.5) | 2120 (30.3) | <.001 |
|  | Poor | 291 (34.0) | 333 (39.0) | 219 (25.6) |  |
|  | Missing | 65 (33) | 80 (41) | 42 (22) |  |
|  |  |  |  |  |  |
| BMI | < 18.5 | 120 (23.5) | 227 (44.5) | 159 (31.2) | <.001 |
| (Kg/m²) | 18.5-25 | 1437 (25.3) | 2461 (43.3) | 1727 (30.4) |  |
|  | 25-30 | 449 (28.8) | 661 (42.5) | 424 (27.2) |  |
|  | > 30 | 52 (32) | 64 (40) | 44 (27) |  |
|  | Missing | 63 (45) | 43 (31) | 27 (19) |  |
|  |  |  |  |  |  |
| Diseases under treatment | No | 794 (24.4) | 1441 (44.3) | 990 (30.4) | .001 |
|  | Yes | 1220 (27.7) | 1843 (41.8) | 1295 (29.4) |  |
|  | Missing | 107 (27.5) | 172 (44.7) | 96 (25) |  |
|  |  |  |  |  |  |
| Depression | No | 1528 (24.6) | 2697 (43.4) | 1930 (31.1) | <.001 |
|  | Yes | 470 (31.9) | 612 (41.6) | 370 (25.1) |  |
|  | Missing | 123 (33.8) | 147 (40.4) | 81 (22) |  |
|  |  |  |  |  |  |
| Population density | Metropolitan | 702 (19.8) | 1504 (42.4) | 1306 (36.8) | <.001 |
|  | Urban | 709 (30.2) | 1008 (42.9) | 609 (25.9) |  |
|  | Semiurban | 257 (24.8) | 517 (49.9) | 243 (23.5) |  |
|  | Rural | 453 (40.5) | 427 (38.2) | 223 (19.9) |  |

a Both missing and other items from the educational attainment variable were excluded from the verification targets.

Table S5: Interaction

|  |  | Total score (n=4491) | | IADL (n=6993) | | Intellectual activity  (n=6800) | | Social role (n=5639) | |
| --- | --- | --- | --- | --- | --- | --- | --- | --- | --- |
|  |  | RR (95%CI) | *P value* | RR (95%CI) | *P value* | RR (95%CI) | *P value* | RR (95%CI) | *P value* |
| Internet use | No | Ref |  | Ref |  | Ref |  | Ref |  |
| Main effect | Yes | 0.56 (0.42-0.75) | <.001 | 0.44 (0.29-0.65) | <.001 | 0.52 (0.34-0.78) | .002 | 0.68 (0.53-0.87) | .002 |
|  | Missing | 0.92 (0.63-1.35) | .68 | 0.89 (0.54-1.48) | .65 | 0.85 (0.51-1.40) | .51 | 0.64 (0.45-0.91) | .01 |
|  |  |  |  |  |  |  |  |  |  |
| Educational attainment | ≤ 9 | Ref |  | Ref |  | Ref |  | Ref |  |
| Main effect | 10–12 | 0.78 (0.62-1.00) | .05 | 0.58 (0.41-0.81) | .001 | 0.50 (0.35-0.72) | <.001 | 0.79 (0.64-0.97) | .026 |
|  | ≥ 13 | 0.86 (0.65-1.13) | .27 | 0.75 (0.52-1.09) | .13 | 0.45 (0.29-0.71) | .001 | 0.77 (0.57-1.04) | .09 |
|  |  |  |  |  |  |  |  |  |  |
| Interaction | No/≤ 9 | Ref |  | Ref |  | Ref |  | Ref |  |
|  | Yes/10–12 | 1.33 (0.92-1.93) | .13 | 1.18 (0.69-2.01) | .55 | 1.54 (0.91-2.61) | .11 | 1.14 (0.86-1.52) | .36 |
|  | Yes/≥ 13 | 1.19 (0.81-1.73) | .38 | 1.10 (0.65-1.87) | .73 | 1.15 (0.61-2.16) | .67 | 1.24 (0.92-1.67) | .16 |
|  | Missing/≤ 9 | Ref |  | Ref |  | Ref |  | Ref |  |
|  | Missing/10–12 | 0.83 (0.48-1.45) | .52 | 0.96 (0.44-2.10) | .92 | 0.28 (0.06-1.30) | .10 | 1.04 (0.62-1.77) | .87 |
|  | Missing/≥ 13 | 0.96 (0.52-1.77) | .89 | 0.73 (0.28-1.93) | .52 | 1.22 (0.38-3.92) | .74 | 1.73 (0.84-3.56) | .14 |
